# Supplementary material for: Heparin-based hydrogel scaffolding alters the transcriptomic profile and increases the chemoresistance of MDA-MB-231 triple-negative breast cancer cells
Source: Biomater Sci. 2020 Feb 13;8(10):2786–96. doi: 10.1039/c9bm01481k (PMC7497406; doi:10.1039/c9bm01481k)
Supplement: Supplementary file 2 [file BM-008-C9BM01481K-s002.zip › Supplementary File 4/HP-BhydrogelvControl/Pathways/my_analysis.Gsea.1545201079907/HALLMARK_ANGIOGENESIS.html]

Details for gene set HALLMARK\_ANGIOGENESIS[GSEA]

|  || Dataset | expr.class.cls#HYD\_versus\_CONTROL.class.cls#HYD\_versus\_CONTROL\_repos |
| Phenotype | class.cls#HYD\_versus\_CONTROL\_repos |
| Upregulated in class | HYD |
| GeneSet | HALLMARK\_ANGIOGENESIS |
| Enrichment Score (ES) | 0.41246384 |
| Normalized Enrichment Score (NES) | 1.208964 |
| Nominal p-value | 0.22278057 |
| FDR q-value | 0.42310166 |
| FWER p-Value | 1.0 |
Table: GSEA Results Summary

  

Fig 1: Enrichment plot: HALLMARK\_ANGIOGENESIS      
 Profile of the Running ES Score & Positions of GeneSet Members on the Rank Ordered List

  

| PROBE | DESCRIPTION (from dataset) | GENE SYMBOL | GENE\_TITLE | RANK IN GENE LIST | RANK METRIC SCORE | RUNNING ES | CORE ENRICHMENT || 1 | PDGFA | na |  |  | 484 | 2.361 | 0.0985 | Yes |
| 2 | VAV2 | na |  |  | 1093 | 1.807 | 0.1615 | Yes |
| 3 | FGFR1 | na |  |  | 1365 | 1.643 | 0.2334 | Yes |
| 4 | JAG2 | na |  |  | 1375 | 1.640 | 0.3189 | Yes |
| 5 | LRPAP1 | na |  |  | 1705 | 1.488 | 0.3797 | Yes |
| 6 | COL5A2 | na |  |  | 2317 | 1.233 | 0.4125 | Yes |
| 7 | VTN | na |  |  | 5473 | 0.557 | 0.2771 | No |
| 8 | PGLYRP1 | na |  |  | 6956 | 0.365 | 0.2189 | No |
| 9 | TNFRSF21 | na |  |  | 9191 | 0.127 | 0.1091 | No |
| 10 | CCND2 | na |  |  | 9609 | 0.088 | 0.0919 | No |
| 11 | SERPINA5 | na |  |  | 9913 | 0.052 | 0.0789 | No |
| 12 | PTK2 | na |  |  | 10391 | 0.008 | 0.0544 | No |
| 13 | THBD | na |  |  | 10820 | -0.042 | 0.0343 | No |
| 14 | FSTL1 | na |  |  | 13295 | -0.325 | -0.0777 | No |
| 15 | VCAN | na |  |  | 13320 | -0.327 | -0.0618 | No |
| 16 | NRP1 | na |  |  | 13560 | -0.362 | -0.0553 | No |
| 17 | APP | na |  |  | 13765 | -0.397 | -0.0451 | No |
| 18 | TIMP1 | na |  |  | 14560 | -0.499 | -0.0604 | No |
| 19 | LPL | na |  |  | 14944 | -0.553 | -0.0514 | No |
| 20 | JAG1 | na |  |  | 15843 | -0.713 | -0.0609 | No |
| 21 | STC1 | na |  |  | 16195 | -0.787 | -0.0380 | No |
| 22 | ITGAV | na |  |  | 16980 | -0.980 | -0.0275 | No |
| 23 | S100A4 | na |  |  | 19100 | -2.731 | 0.0051 | No |
Table: GSEA details [plain text format]

  

Fig 2: HALLMARK\_ANGIOGENESIS      
 Blue-Pink O' Gram in the Space of the Analyzed GeneSet

  

Fig 3: HALLMARK\_ANGIOGENESIS: Random ES distribution      
 Gene set null distribution of ES for **HALLMARK\_ANGIOGENESIS**
